# Dendritic Cell-Based Vaccines Positively Impact Natural Killer and Regulatory T Cells in Hepatocellular Carcinoma Patients

**DOI:** 10.1155/2011/249281

**Published:** 2011-09-28

**Authors:** Sarah M. Bray, Lazar Vujanovic, Lisa H. Butterfield

**Affiliations:** ^1^Departments of Medicine, University of Pittsburgh Cancer Institute, Pittsburgh, PA 15213, USA; ^2^Department of Surgery, University of Pittsburgh Cancer Institute, Pittsburgh, PA 15213, USA; ^3^Department of Immunology, University of Pittsburgh Cancer Institute, Hillman Cancer Center, Pittsburgh, PA 15213, USA

## Abstract

Immunotherapy of cancer must promote antitumor effector cells for tumor eradication as well as counteract immunoregulatory mechanisms which inhibit effectors. Immunologic therapies of cancer are showing promise, including dendritic cell-(DC-) based strategies. DC are highly malleable antigen-presenting cells which can promote potent antitumor immunity as well as tolerance, depending on the environmental signals received. Previously, we tested a peptide-pulsed DC vaccine to promote Alpha-fetoprotein (AFP-) specific anti-tumor immunity in patients with hepatocellular carcinoma (HCC), and reported on the CD8^+^ T cell responses induced by this vaccine and the clinical trial results. Here, we show that the peptide-loaded DC enhanced NK cell activation and decreased regulatory T cells (Treg) frequencies in vaccinated HCC patients. We also extend these data by testing several forms of DC vaccines *in vitro* to determine the impact of antigen loading and maturation signals on both NK cells and Treg from healthy donors and HCC patients.

## 1. Introduction

Hepatocellular carcinoma (HCC) is the third leading cause of cancer mortality worldwide [1]. It often follows cirrhosis caused by viral or alcoholic hepatitis. Prognosis remains very poor, and treatment options are few [1]. Curative surgery and liver transplantation are only available to a small minority of early-stage HCC patients. Other common therapies (including ablative therapies and Sorafenib) are largely palliative. Treatment is complicated by preexisting cirrhosis, as chemotherapy or resection may not be options in a patient with poor liver reserves. 

Alpha-fetoprotein (AFP) is an oncofetal antigen that is expressed by more than half of HCC tumors and detectable at elevated levels in the blood and tumor microenvironment in these HCC patients [2]. AFP serves as the most common serum biomarker for HCC and, as it is from undetectable to 10 ng/mL in healthy adults [3], has also been identified as a specific tumor-associated antigen for HCC immunotherapy [4]. We and others have investigated AFP as a tumor rejection antigen for immunotherapy of HCC [5–13]. 

Dendritic cell (DC) vaccines are promising vehicles for activating antitumor specific T cells and NK cells for tumor immunotherapy. They are immunologic sentinels which can induce antigen-specific immunity or tolerance [14, 15]. DC can be activated or matured with cytokines and toll-like receptor (TLR) agonists such as interferon gamma (IFN*γ*) or lipopolysaccharide (LPS) [16–19]. Antigen loading of DC can be achieved in a number of ways, including peptide pulsing, whole protein loading, and genetic engineering via viral transduction. 

While DC are critical to induction of immunity, other immune cells are important as effectors and regulators in cancer immunity. CD56^+^CD16^+/-^ natural killer (NK) cells are the effectors of the innate immune system that are able to directly kill tumor or virally infected cells with reduced levels of MHC class I molecules or that overexpress stress-induced activating cell surface molecules (e.g., MICA/B recognition via NKG2D), and that otherwise may escape immune detection. Hepatic lymphocytes are enriched (up to 30%) in NK cells that may play a role in antitumor defense [20]. Murine models support a role for NK cell effectors in liver tumor responses. In mice, antitumor effects mediated by NK cells were IFN-*γ* dependent [21]. The CD4^+^CD25^hi^FOXP3^+^ T regulatory (Treg) cell has more recently been recognized as an important target in immunotherapy because of its role in inhibiting the immune response. Patients with HCC have been shown to have defects in NK cell function [22] and high intratumoral [23] and circulating levels of Treg [24], all of which may impact the progression of this disease.

We previously tested an AFP peptide-pulsed DC vaccine in a phase I clinical trial. The vaccine was found to be safe and immunogenic in late-stage HCC patients [25–27]. We detected type I immunity induced to the 4 immunizing HLA-A*0201-restricted AFP-derived peptides in the majority of patients by IFN-*γ* ELISPOT and MHC class I tetramer assays.

It has been demonstrated that DC and NK cells are capable of interacting with and activating each other [28–30]. We have found that recombinant adenovirus (AdV)-transduced DC (AdV/DC), unlike immature DC, are capable of functionally activating NK cells [17]. There are also circumstances in which DC can promote Treg expansion. In this study, we examined the *in vivo* effects of AFP peptide-pulsed DC on NK cell activation and Treg frequencies and phenotypes in peripheral blood mononuclear cells (PBMC) of HCC patients and described evidence for both NK cell activation and decreased frequencies of FOXP3^+^ Treg cells. We then compared several clinically relevant DC preparations for effects on NK cells and Treg *in vitro* and find differences in the DC groups and between HCC patients and healthy donors (HD). We show that AdV/DC, with (pmAdV/DC) or without maturation, are most successful at inducing NK cell activation and Treg depletion. The results have relevance for the design DC-based vaccines in patients with HCC.

## 2. Materials and Methods

### 2.1. Patient and Healthy Donor Cells

PBMC were obtained from healthy volunteers (HD) and from HCC patients enrolled in a peptide-pulsed DC vaccine (UCLA IRB #00-01-026, IND BB9395; UPCI #04-001 and #04-111; informed consent was obtained from all patients and donors). The clinical trial was previously published in detail [26] which included immunologic monitoring of vaccine responses from banked PBMC. Limited patient data is listed in Table 1. PBMC were isolated using a Ficoll gradient and either tested fresh (some HD) or were cryopreserved (some HD and all HCC patient cells) in RPMI1640/20% human AB serum/10% DMSO for later testing.

### 2.2. Flow Cytometry

Cells were stained according to manufacturer recommendations, fixed in 0.5% paraformaldehyde, and analyzed on an CyAn high-speed analyzer (Dako, Carpinteria, Calif) (UPCI Flow Cytometry Facility) and the Summit v4.3 software within four days. NK cell phenotype was investigated using: CD8 PE, CD16 ECD, CD3 APC (Beckman Coulter), granzyme B FITC, CD25 PE-Cy7, CD56 PE-Cy5, and CD69 APC-Cy7 (BD Pharmingen). Treg were investigated using: CD4 FITC, FOXP3 PE, and CD25 APC (eBioscience) and reported as either the FOXP3 positive percentage or the MFI of FOXP3 expression in the CD4^+^CD25^+^ cells.

### 2.3. Cell Isolation, DC Growth, and Vaccine Models (See Figure 1)

CD56^+^ NK cells and CD4^+^ T cells were isolated from PBMC (Miltenyi Biotech) according to the manufacturer's directions (CD56 beads, NK isolation kit, CD4^+^ T cell isolation kit). Change in MFI was considered “positive” if the increase was ≥25% of the baseline MFI. Percent positivity was considered positive if ≥5% greater than baseline.

Monocytes were isolated from PBMC using adherence to T75 flasks (Costar). They were cultured for 6-7 days in RPM1640/5% human AB serum/PennStrep medium with 500 U/mL IL-4 and 800 U/mL GM-CSF (Schering-Plough, Kenilworth, NJ; Amgen, Thousand Oakes, Calif) to promote differentiation to myeloid DC. 

After culture, DC were harvested, counted (Trypan Blue Stain; BioWhittaker, Walkersville, MD), and cultured as described below. DC were subsequently cocultured with NK cells or T cells isolated from the autologous donor and incubated 24 hr (NK and CD4) to 5 days (CD4) at ratios of 1 DC to 1–10 NK or T cells. After the coculture, cells were harvested, supernatant was collected and stored at −80°C, and cells were analyzed by flow cytometry as described above. 

For peptide-pulsed DC (pep/DC), DC were pulsed with 1 or 2 specific AFP peptides (AFP_158_ FMNKFIYEI and AFP_542_ GVALQTMKQ; synthesized at the University Pittsburgh Peptide Synthesis Facility) at 10 *μ*g/mL for 2 hr at 37°C, then washed in medium before further culture. Similarly, for protein-pulsed DC (prot/DC), DC were loaded with cord blood-derived hAFP protein (CalBiochem) at 10 *μ*g/mL for 2 hr at 37°C, then washed. 

For AdV-transduced DC (AdV/DC), DC were transduced for 2 hr at 37°C in serum-free media (IMDM) at MOI = 1,000 with an AdV encoding full length AFP protein (AdVhAFP) [5] and then washed in medium before further culture. In the case of “prematured” AdV-transduced DC (pmAdV/DC), DC were first matured 24 hr with 250 ng/mL LPS (Sigma) and 1000 U/mL IFN-*γ* (Pepro Tech), after which they were washed and transduced with AdVhAFP as described above.

### 2.4. Luminex

Cell-free supernatants were collected from cultures and frozen at −80°C. They were subsequently thawed and simultaneously analyzed with the multiplex Luminex assay (Invitrogen) per manufacturer's protocol in a BioRad reader (UPCI Immunologic Monitoring Laboratory). The following analytes were tested: GM-CSF, IFN-*γ*, IP-10, MCP-1, TNF-*α*, IL-1*β*, IL-2, IL-4, IL-5, IL-6, IL-8, and IL-10 in a kit pretested for any potential crossreactivity by the manufacturer. Controls included the included standard curve and multiplex QC standards (R&D Systems).

### 2.5. Statistical Analysis

One-tail *t*-test analyses were used to estimate statistical significance of differences obtained; *P* values ≤0.05 were considered to be statistically significant.

## 3. Results

Based on our previous study (17), we hypothesized that HCC patients vaccinated with immature DC pulsed with AFP peptides (pep/DC) would not impact activation of circulating NK cells. We assessed this by evaluating upregulation of CD69 or CD25 activation markers on CD56^hi^/CD16^−^ and/or CD56^lo^/CD16^+^ NK cell subsets. We also wished to determine whether Treg frequency (as determined by a change in FOXP3-expressing CD4^+^CD25^hi^ T cells) was modulated by vaccination, which might also include changes in CTLA-4 [31, 32] or CCR7 [33] expression on Treg. 

### 3.1. HCC Patients Treated with AFP Peptide-Pulsed DC Exhibit Elevated Levels of NK Cell Activation

**Figure 1:**
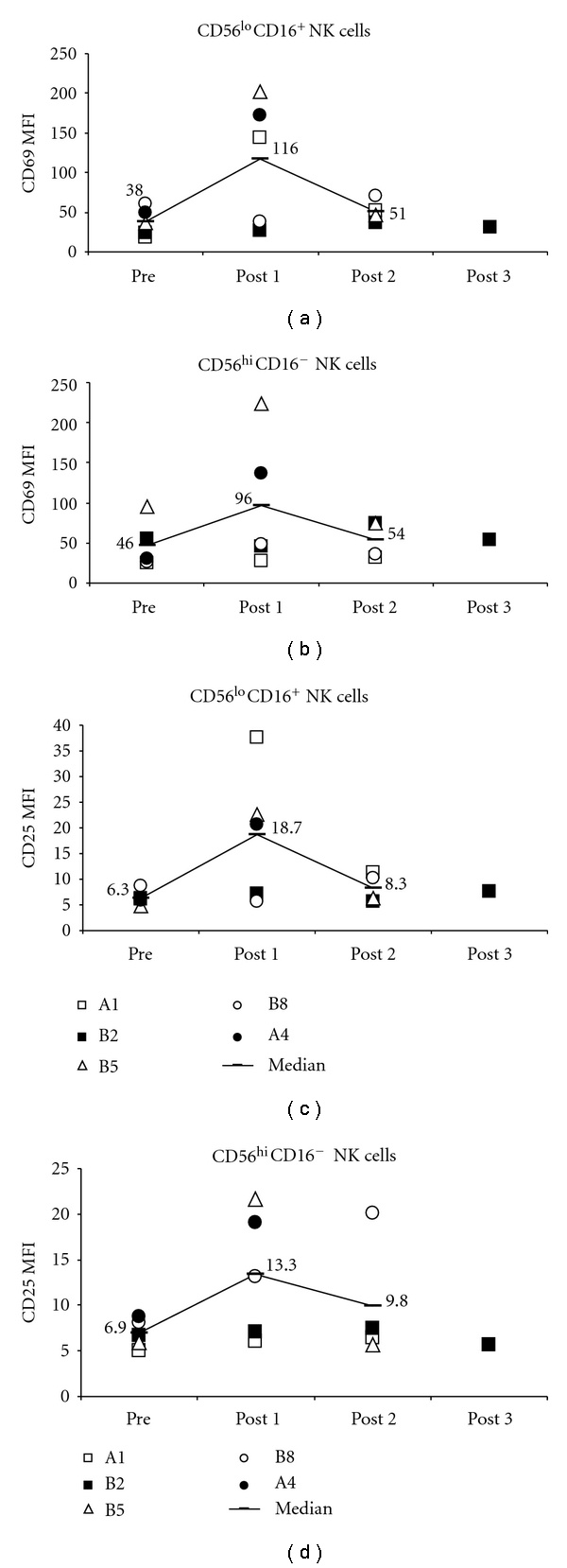
CD69 and CD25 expression on CD56^lo^CD16^+^ and CD56^hi^CD16^−^ NK cells. Phenotyping of NK cells from patients who received the AFP pep/DC vaccine, showing longitudinal changes. “Pre” denotes PBMC from time point 0. DC vaccines were delivered (after blood draws) on days 0, 14, and 28. “Post” denotes PBMC from postvaccine administration at time points available in the remaining batched PBMC. Patient A1 tested at days 35 and 56 (7 days and 28 days after the third vaccine); pt B2 at d28, 56, and 112; pt B5 at d14 and 28; pt B8 and d14 and 112, pt A4 at d14. CD69 and CD25 markers (by MFI) are shown for both NK cell subsets. Percent positivity is shown in Supplementary Figure  2. One-tail *t*-test *P* values are: CD56^lo^CD16^+^CD69 MFI: pre to post 1: 0.05; pre to post 2: 0.03; post 1 to post 2: 0.16; all other values higher and not significant (n.s.). CD56^hi^CD16^−^CD69 MFI: pre to post 1: 0.07, all other values higher and n.s. CD56^lo^CD16^+^CD25 MFI: pre to post 1: 0.05, pre to post 2: 0.11, post 1 to post 2: 0.12, all other values higher and n.s. CD56^lo^CD16^−^CD25 MFI: pre to post 1: 0.04, pre to post 2: 0.15, all other values higher and n.s.

We tested banked PBMC samples from five HCC patients, isolated at different time points during vaccination with AFP pep/DC. Cells were stained immediately after thawing to assess phenotype by flow cytometry (see analysis strategy shown in Supplemental Figure  1 in Supplementary Material available online at doi:10.1155/2011/249281). Contrary to our hypothesis, both regulatory CD56^hi^CD16^−^ and cytotoxic CD56^lo^CD16^+^ NK cells demonstrated activation post-pep/DC vaccination, compared to baseline. Activation was determined by both an increase in population MFI (Figure 1) as well as percent positivity for CD25 or CD69 (Supplemental Figure  2). CD69 expression was increased in 4/5 patients for both CD56^lo^CD16^+^ and CD56^hi^CD16^−^ NK cells according to MFI and percent positive values (Figure 1 and Supplemental Figure  2). CD25 expression was also increased by percent positivity and MFI in 3/5 and 4/5 patients' CD56^lo^CD16^+^ NK cells (Figure 1). For CD56^hi^ NK cells, CD25 was increased in 4/5 patients by MFI only. Patient B2 (CD56^lo^CD16^+^CD69 percentage and CD56^hi^CD16^−^CD69 MFI) and B8 (CD56^lo^CD16^+^CD69 and CD25 by both MFI and percentage) had a decrease in NK cell activation after first vaccination, with an increase after second immunization. The other patients displayed stronger NK cell activation. Of the two main NK cell subsets, CD56^lo^CD16^+^ cells showed the greater degree of activation. Patients A1, A4, and B5, with multiple time point samples available, had highest NK cell activation after the first round of vaccination, with subsequent time points showing less activation. Additionally, we tested these cells for the expression of NKG2D, an NK cell activating receptor; however only low levels of this molecule were detected on a small percentage of circulating NK cells (data not shown).

### 3.2. HCC Patients Treated with AFP Peptide-Pulsed DC Vaccines Display Decreased Frequencies of Circulating Treg Cells

**Figure 2:**
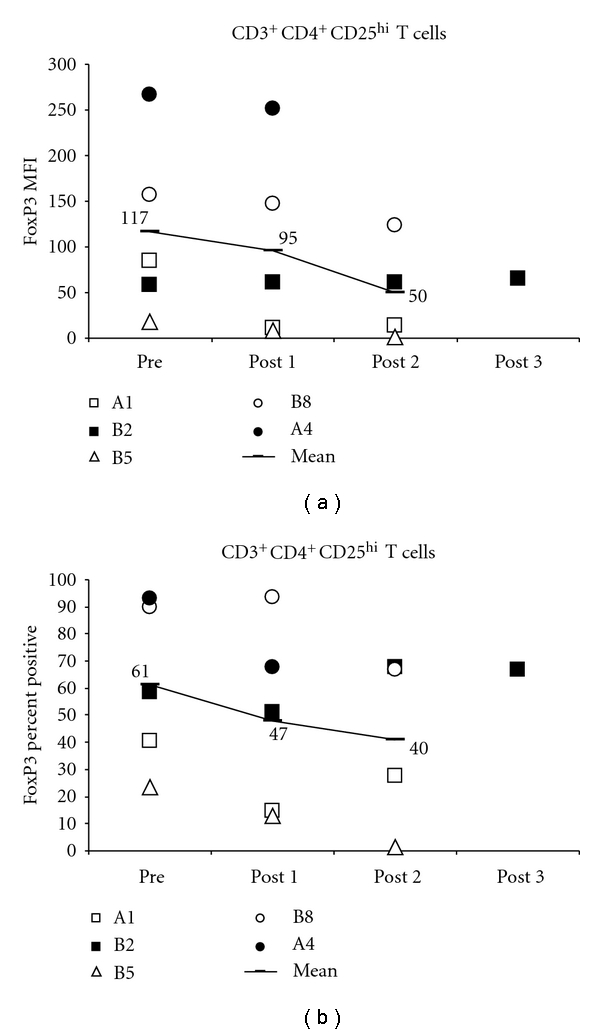
FOXP3 expression in CD4^+^CD3^+^CD25^hi^ (Treg) cells. Phenotyping of Treg cells from patients who received the pep/DC vaccine, showing longitudinal changes. “Pre” denotes PBMC from time point 0. “Post” denotes PBMC from postvaccine administration at time points available. Patient A1 tested at days 35 and 56; pt B2 at d28, 56, and 112; pt B5 at d14 and 28; pt B8 and d14 and 112, pt A4 at d14. (a) FOXP3 assessed intracellularly in CD3^+^CD4^+^CD25^hi^ cells, showing MFI, or (b) % positivity. One-tail *t*-test *P* values are: FOXP3 MFI: pre to post 1: 0.09, pre to post 2: 0.07, post 1 to post 2: 0.17; all other values higher and n.s. FOXP3 percent positive: pre to post 1: 0.03; pre to post 2: 0.10; all other values higher and n.s.

To examine Treg cell frequencies, CD3^+^CD4^+^ T cells were gated on CD25^hi^ or total CD25^+^ and intracellularly stained for FOXP3. The Treg lymphocyte frequencies were then assessed by flow cytometry. FOXP3 expression in the CD3^+^CD4^+^CD25^hi^ T cells showed a consistent change, decreasing overall in 4/5 of the patients tested, by both percent positivity and MFI (Figure 2). Similar to the NK cell activation measures, patient B2 also showed inferior Treg changes by either measure. Treg frequencies slightly increased in this patient after second and third immunizations. CD3^+^CD4^+^CD25^hi^ cell surface CCR7 and CTLA-4 showed minor variation over time and were not considered informative in our data set (data not shown).

### 3.3. Phenotypic Changes in DC with Different Antigen Loading Strategies

The AFP peptide-pulsed DC did not undergo a specific maturation step during vaccine preparation. Maturation cocktails can impact surface levels of MHC class I and II, costimulatory molecule levels, and cytokine production. We hypothesized that different DC antigen-loading strategies, some of which impact DC maturation, would result in unique phenotypic changes in the DC that would impact activation and frequencies of other immune cells (like NK cells and Treg) they interacted with. We previously tested AdV-mediated genetic engineering of DC to enable expression of full length antigens in DC [6, 34–37], and found that AdV transduction promotes partial maturation of DC and superior antigen-specific T cell responses. Since we have identified AFP as a tumor-associated T cell antigen for HCC [5, 10], we utilized the AFP antigen in multiple forms (AdVhAFP, AFP protein, AFP-derived peptide) for DC loading. We transduced HD DC with AdVhAFP and compared them to immature DC (iDC) and LPS/IFN-*γ*-matured DC (mDC). DC groups were then cocultured with autologous NK or CD4^+^ T cells to determine the impact on these lymphocytes. We examined CD83, CCR7, CD86, and CD80 as markers of DC maturation. We found that all four markers were upregulated after AdV transduction as compared to iDC, but that their greatest upregulation was observed in mDC (data not shown, similar to [19, 37]). We also found that coculture with resting NK cells modestly improved the expression of these DC maturation markers in comparison to the level of modulation achieved by AdV transduction or LPS/IFN-*γ* treatments alone (data not shown, similar to [17]).

### 3.4. Changes in NK Cell Activation Levels and Treg Frequencies after Culture with DC

We hypothesized that NK cells would be activated (as measured by increased CD69 and CD25 expression) and that Treg frequencies might be reduced (decreased FOXP3 expression) after interactions with DC that were at least partially matured and that these trends would be observed after coculture with the AdV/DC. Monocyte-derived DC were antigen-loaded as described and cocultured with autologous NK or CD4^+^ cells for 24 hr or 6 days, respectively. The cells were then harvested and assessed by FACS for phenotypic changes in specific subsets. 

**Figure 3:**
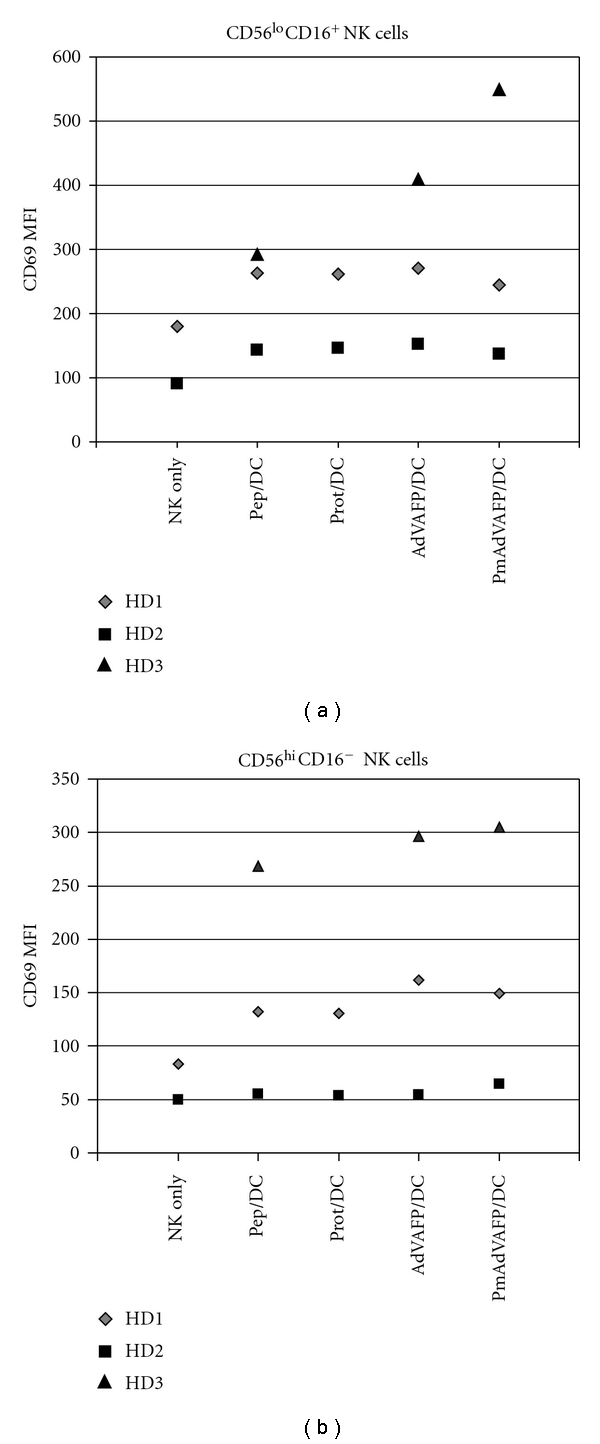
CD69 expression on healthy donor CD56^hi^ and CD56^lo^CD16^+^ NK cells. Phenotyping of NK cells from HD PBMC after 48 hr coculture with different DC groups, showing CD69 upregulation on the (a) CD56^lo^CD16^+^ and (b) CD56^hi^CD16^−^ subsets for three HD.

We first assessed different AFP antigen loading modalities (peptide, protein and AdV) in HD cells. Peptide pulsing and protein-loading do not include any maturation agents and have been observed not to alter DC phenotype *in vitro* while AdV provides a partial maturation signal from the viral transduction. While we recently investigated the ability of AdVLacZ-transduced DC to interact with NK cells in depth [17], the present study focused on AFP, which has been reported to have immune suppressive functions [38, 39]. In line with these publications, we have observed that AdVhAFP/DC express less transmembrane TNF than AdVLacZ/DC (L. Vujanovic and LH Butterfield, unpublished data, 2011). Here, we confirmed that NK cells cocultured with DC upregulate CD69 (increase in MFI values (Figure 3(a)) and percent positivity (not shown)). Activation at 48 hr was somewhat stronger than at 24 hr (not shown). Sufficient HD cells were available to also test “pre-matured” (first matured with IFN-*γ* and LPS) then AdV-transduced DC (pmAdVhAFP/DC), which we found to more potently activate antigen-specific CD8^+^ T cells than AdV/DC alone [19]. Overall, DC transduced with AdV and/or pre-matured and AdV-transduced were slightly more potent NK cell activators than pep/DC or prot/DC (which were similar to each other). 

**Figure 4:**
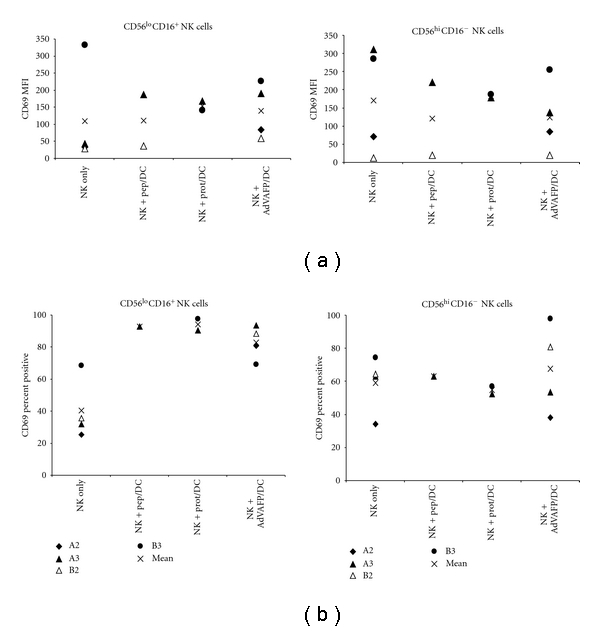
CD69 expression on HCC patient NK cells. Phenotyping of NK cells from HCC patients after 48 hr coculture with different groups of DC, showing CD69 upregulation on CD56^hi^CD16^−^ and CD56^lo^CD16^+^ subtypes, by MFI (a) and percent positivity (b).

We then tested the impact of differentially antigen loaded and matured DC on NK cell activation of HCC patients. HCC patient CD56^lo^CD16^+^ NK cells showed increased level of activation (CD69 MFI and percent positivity) after coincubation with DC (3/4 patients, particularly with AdVhAFP/DC, Figure 4). However, the CD56^hi^CD16^−^ NK cells from HCC patients minimally (if at all) were activated by the different DC groups. Two of the patients (A3, B3) had CD56^hi^CD16^−^ NK cells which (according to MFI values) expressed high levels of CD69 without activation (also higher than the levels detected in other patients, Figure 1(a)), which were not further activated by DC. Overall, patient CD56^lo^CD16^+^ NK cells showed the ability to increase in CD69 expression, particularly after coincubation with AdV/DC as compared to NK alone, and other antigen-loaded DC produced more variable responses (Figure 4). 

**Figure 5:**
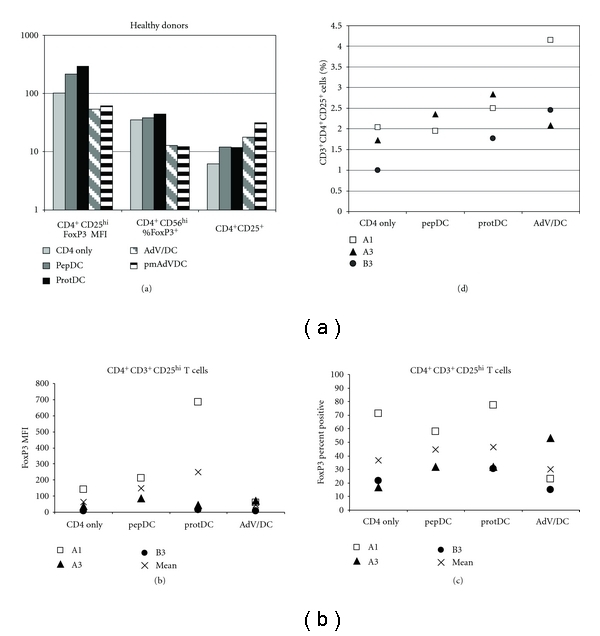
Treg cell responses to DC coculture. HD (a) and HCC patient (b, c, and d) CD4^+^ T cells were cocultured with differentially treated DC. The FOXP3 expression in CD3^+^/CD4^+^/CD25^hi^ cells is shown in (a) as MFI (left), percent positive (middle). The right group is the frequency of total activated (CD25^+^) CD4^+^ T cells. The FOXP3 MFI in Treg in HCC patients is shown in (b). The percent CD3^+^/CD4^+^/CD25^hi^/FOXP3^+^ Treg in HCC patients is shown in (c). The overall frequency of activated CD4^+^ T cells is shown in (d) (% CD3^+^/CD4^+^/CD25^+^ cells).

In order to determine any impact of DC antigen loading on Treg expansion *in vitro*, HD CD4^+^T cells were cocultured for 6 days with different DC preparations. The pep/DC and prot/DC-stimulated groups showed increased CD3^+^CD4^+^CD25^hi^FOXP3^+^ cells, while both AdVhAFP/DC and pmAdVhAFP/DC-stimulated groups showed reduced Treg frequencies and FOXP3 expression levels, compared to baseline levels (Figure 5(a)). The opposite pattern was seen with the total CD3^+^CD4^+^CD25^+^-activated T cell population, indicating that the AdV/DC were superior for overall activation of CD4^+^ T cells (the frequency of CD3^+^/CD4^+^/CD25^+^ cells), without expanding FOXP3^+^ Treg. Using the same experimental method, three HCC patient cells were tested for Treg expansion. While patients A1 and B3 showed the same pattern as the HD, HCC patient A3 showed a different pattern, in which no DC group showed a relative reduction in Treg *in vitro* (Figures 5(b) and 5(c)). The total CD3^+^CD4^+^CD25^+^-activated cells were tested in the patient cultures, and patients A1 and B3 again showed the same pattern as the HD, while patient A3 showed most activated cells in the prot/DC group (Figure 5(d)).

### 3.5. Cytokine Production

**Figure 6:**
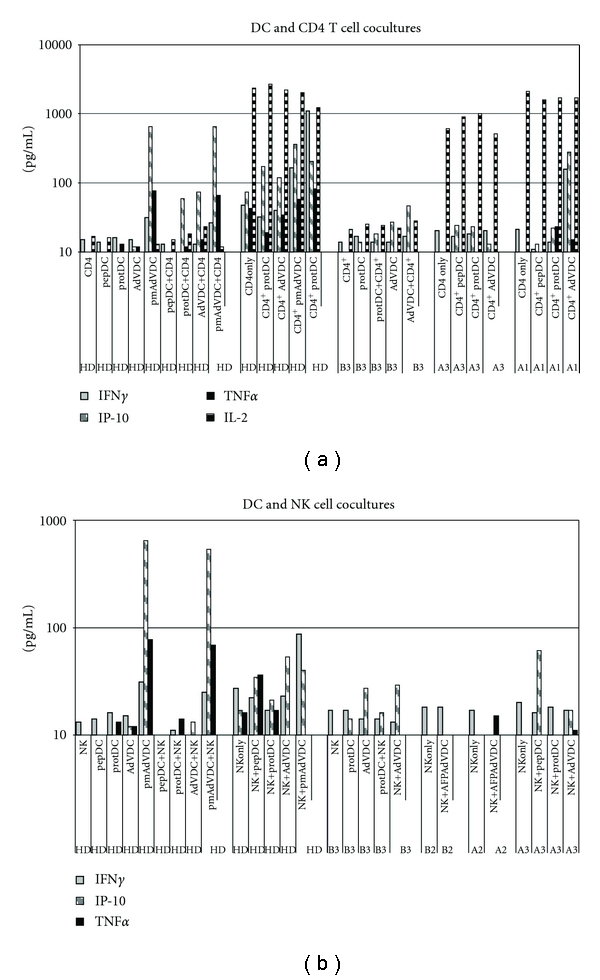
Luminex results: production of chemokines and cytokines. Graphs are grouped according to scale of cytokine production and function. (a) IFN-*γ*, IP-10, TNF-*α*, and IL-2 production after CD4^+^ DC coculture. (b) MCP-1 and IL-5 production after CD4^+^ DC co-culture.

In order to characterize the DC-lymphocyte (NK cell or CD4^+^ T cell) interaction environment, cell-free supernatants were collected from the different HD and HCC patient cell coculture experiments. Supernatants were tested by multiplex Luminex assay to simultaneously assess the levels of cytokines, chemokines and growth factors, including: GM-CSF, IFN-*γ*, MCP-1, TNF-*α*, IL-1*β*, IL-2, IL-4, IL-5, IL-6, IL-8, IL-10, and IP-10 (Figure 6 and not shown). This allowed us to examine a functional readout of those interactions. IL-10 (produced by suppressive cells in some settings) was largely below the level of detection (5 pg/mL). Of the “Th1/effector” cytokines and chemokines in the cocultures of CD4^+^ T cells and DC groups (IFN-*γ*, IP-10, TNF-*α*, IL-2), IFN-*γ* was notably higher in the two HD and one HCC (A1) coculture of CD4^+^ with AdV/DC, as compared to the other DC conditions. IP-10 also showed an increase for CD4^+^ T cells stimulated with AdV/DC (both HD and two patients: A1 and B3). Additionally, elevated IP-10 secretion was observed in prot/DC^+^CD4^+^ T cell cultures of HD1 and all three patients, but not to the level observed in AdV/DC cocultures. IL-2 expression, detected in all patients and only one HD, was only minimally influenced by DC and appeared CD4^+^ T cell derived (data not shown). HD IFN-*γ*, IP-10, and TNF-*α* production was much higher than HCC patient levels (Figure 6(a)). Similar to TNF-*α* and IP-10, MCP-1 expression was greater in HD and was largely derived from immature DC and AdV/DC (reduced in pmAdV/DC, not shown). IL-5 expression was detected in one HD and two HCC cell co-culture of CD4^+^ with AdV/DC (not shown). 

Coculture of NK cells with the differently antigen-loaded DC groups yielded minimal levels of IFN-*γ* and TNF-*α* and IP-10 which were largely restricted to HD cells and pmAdV/DC (Figure 6(b)). As above with the CD4^+^ T cell cocultures, MCP-1 production was robust in all co-cultures and DC derived (not shown). IL-6 was restricted to HD and pmAdV/DC cocultures (not shown). Lastly, IL-8 were broadly detected in most groups and were more highly expressed in HD cultures than HCC cultures (not shown). These data highlight important functional differences between HD and HCC cells, with reduced cytokine and chemokine production levels (but some similar trends) among the HCC patients.

## 4. Discussion

Immunotherapy holds potential for treatment of hepatocellular carcinoma, as few effective treatments are available, and immunotherapy vaccine strategies have largely shown immunogenicity and less toxicity than current chemotherapy [40–44]. We have previously conducted vaccination clinical trials of AFP-based vaccines for HCC, with a goal of activating the immune system against cells expressing the AFP oncofetal antigen. Our current investigation was undertaken with two aims. First, we sought to define phenotypic changes in NK cells and Treg over time in the patients treated with an AFP pep/DC vaccine, for the first time. Second, we tested *in vitro* responses of NK cells and Treg to DC vaccines including peptide pulsed, protein-loaded, and AdV-transduced, to determine the DC antigen loading and maturation strategy which would promote NK activation and minimize Treg expansion. Such data are critical for the design of next generation DC vaccines with broad immunologic impact on both effectors and suppressive mechanisms. 

An effective vaccine against HCC would activate not only tumor antigen-specific adaptive immune responses, but also innate NK cell effectors to crosstalk with DC, promote type I responses, and potentially also directly kill HCC cells. In addition, downregulating Treg cells would help to minimize immunosuppression and potentially allow enhanced antitumor effector function. By testing PBMC from the peripheral blood of patients treated with the AFP pep/DC vaccine, we found evidence for activation of NK cells in most patients, as shown by increase in CD69 and CD25 expression. We also found evidence for downregulation of Treg cells in most patients, as shown by decreased FOXP3 expression in those CD4^+^CD25^+^ T cells. These results illustrate the possibility of rationally modulating the immune system with DC to increase anti-HCC immunity. While additional functional assays of NK cell killing and Treg suppression would have strengthened our report, there were insufficient banked PBMC remaining for such assays.

In this data set, A1, A4, and B2 received 10^6^ DC/vaccine, and B5 and B8 received 5 × 10^6^ DC/vaccine, and all were stage IV (Table 1). Because A1 showed both NK cell activation and Treg FOXP3 decrease and B2 showed neither, there does not appear to be an overt DC dose effect for these assays. A1, B5, and B8 were heavily pretreated with a variety of chemotherapies, while B2 had only surgery before the DC vaccines, hence the chemotherapies do not appear to absolutely preclude NK cell and Treg changes observed [26].

By testing *in vitro* responses to a variety of DC vaccines, we were able to assess their comparative ability to stimulate NK and Treg cells. We found that of the antigen-loading strategies and maturation treatments we tested, AdV/DC tended to activate NK cells more than pep/DC or prot/DC as measured by CD69 expression. In addition, HD cells showed downregulation of FOXP3 in CD4^+^CD25^hi^ Treg cells in the presence of AdV/DC (or pmAdV/DC) as compared with pep/DC or prot/DC. These AdV/DC groups also promoted increased CD3^+^CD4^+^CD25^+^ total activated T cells. When testing DC groups with HCC patient cells, the levels of NK cell activation, and differences between groups, were weaker. While our sample size was small, these data highlight the different outcomes from advanced stage HCC patients. This may also indicate an NK cell function defect in these advanced stage patients [26], as noted by others [22].The Treg assessments also indicate that the general trends observed in HD samples could be similar to HCC patient cells, but there were weaker responses and exceptions. Overall, these results support our conclusions from the phenotyping of vaccinated HCC patient NK and Treg cells, that the immune system can be favorably modulated in multiple ways by the DC vaccines. Furthermore, they indicate that an AdV/DC vaccine may be superior to peptide or protein loaded DC, although patient-specific differences should be anticipated. In future studies, we will also test pre-matured AdV/DC with patient-derived cells, as our banked samples were in insufficient numbers to test all DC groups in all patients. 

Cytokine production in response to DC vaccine co-culture is a functional measure of activation of NK and Treg cells. It was interesting to note that cytokines and chemokines tested for by Luminex were produced more abundantly by HD-derived cells than HCC-derived cells. This again highlights the difficulty of inducing an antitumor immune response in HCC patients, and suggests that additional immune stimulatory and immune suppression reducing efforts may be required to promote the desired antitumor immunity *in vivo*. The AFP antigen may also play a role in these responses. We have observed similar DC phenotypic maturation effects after AdV transduction regardless of transgene; however, some specific molecular changes have been observed. We recently demonstrated that AdV/DC activate NK cells via transmembrane TNF and trans-presented IL-15 [17]. In order to promote that DC-NK cell contact, we have also found that AdV/DC produce IL-8 and IP-10, which cause chemotaxis of NK cells towards DC (Vujanovic and Butterfield, submitted 2011). In this study, we find that the HD cocultures produced more IL-8 and IP-10 (not shown and Figure 6) than the HCC cocultures.

We have performed additional preliminary studies comparing AdVLacZ and AdVhAFP in HD DC, and we find a reduction in DC surface transmembrane TNF expression (but similar *trans*-presented IL-15) with AdVhAFP. These data support the conclusion that preclinical studies to develop more effective AFP-based immunotherapy approaches for HCC should utilize patient cells, and that additional compensatory manipulations may be required to fully activate NK cells in addition to antigen-specific T cells, while limiting Treg expansion.

## 5. Conclusions

In conclusion, we find that DC-based vaccines can modulate not only antigen-specific T-cell responses, but also innate effectors and counter-regulatory mechanisms. Optimal antigen loading of DC and maturation signaling may allow for development of DC vaccines which will subsequently deliver specific signals to the broad array of tumor-reactive cells they encounter *in vivo*. Immunotherapy of cancer has the potential to improve treatment for many cancers, and this investigation into NK cell activation and Treg modulation induced by DC vaccines against HCC is a step forward for designing the next generation of DC vaccines. 

## Supplementary Material

Supplementary Figure 1: shows dot plot details and the gating strategy used for NK cells and Treg.
Supplementary Figure 2 shows the percent positive data for the groups shown as MFI results in manuscript Figure 1.

## Figures and Tables

**Table 1:** HCC patient demographics.

Pt.	Dose DC	Risk Factor	Stage	Previous treatments^1^	Pre-AFP (ng/mL)	Post-AFP (ng/mL)	Response^2^	PFS^3^ (mo)	OS^4^ (mo)
A1	1 × 10^6^	?	IVb	Chemoembo, CDDP, Adriamycin, 5-FU, Xeloda, Thalidomide	2.811	2.748 (+28)	PD	0	4
A2	1 × 10^6^	HBV	IVa	Chemoembo	4.740	5.770 (d + 28)	PD	0	20
A3^5^	1 × 10^6^	EtOH	IVa	RFA	3.080	(no DC)	(no DC)	0	2
A4^6^	1 × 10^6^	HCV	IVb	—	10,800	10.650	(1 vaccine)	0	—
B2	1 × 10^6^	?	IVa	Surgery	5.100	7.650 (d + 35)	PD	0	4
B3^7^	5 × 10^6^	HCV	IVa	Chemoembo RFA	102	65 (d + 28)	NE	0	35
B5	5 × 10^6^	HBV	IVa	Chemoembo, CarboTaxol, Xeloda	1.630	2.515 (d + 112)	PD	0	3+
B8	5 × 10^6^	HCV	IVb	Chemoembo	96.7	134	PD	0	5+

^1^Previous treatments received (chemoembo, chemoembolization; CDDP, cis-platin; 5-FU, 5-flouro-uracil; Xeloda, capecitabine; RFA, radiofrequency ablation; carbo, carboplatin; XRT, radiation therapy).

^2^PD: progressive disease, NE: no evidence of disease.

^3^PFS: progression free survival.

^4^OS: overall survival.

^5^No DC: no DC vaccines could be generated which passed clinical protocol release criteria.

^6^1 vaccine: patient progressed early and did not receive the 3 DC vaccinations.

^7^NE: patient B3 responded to chemoembolization and RFA and was vaccinated shortly thereafter, and had 35 mo. OS.

## Abbreviations

HCC: hepatocellular carcinoma
DC: dendritic cells
AFP: Alpha-fetoprotein
AdV: adenovirus
MFI: mean fluorescence intensity
Treg: regulatory T cells
PBMC: peripheral blood mononuclear cells.
