# Supplementary material for: Dendritic Cell-Based Vaccines Positively Impact Natural Killer and Regulatory T Cells in Hepatocellular Carcinoma Patients
Source: Clin Dev Immunol. 2011 Sep 28;2011:249281. doi: 10.1155/2011/249281 (PMC3182577; doi:10.1155/2011/249281)
Supplement: Supplementary file 1 — Supplementary Figure 1: shows dot plot details and the gating strategy used for NK cells and Treg. Supplementary Figure 2 shows the percent positive data for the groups shown as MFI results in manuscript Figure 1. [file 249281.f1.docx]

**Supplementary Figure 1**. Examples of flow cytometric gating strategies for NK cells and Treg. For each cell type, the top left panel indicates the lymphocyte gate; top right panel shows the exclusion of doublets; the bottom left shows specific cell markers gated on, and the bottom right shows (NK cells) histograms of CD69 activation or (Treg) the intracellular FOXP3 staining.

**Supplementary Figure 2**. CD69 and CD25 percent positivity on HCC patient NK cell subsets. The cell samples in figure 1 (and other experiments) were also analyzed for percent positivity for each marker, based on flow cytometric markers set to negative populations. Figure 1 shows the total specific cell population MFI, and the corresponding percent positive readout is shown here.
